# Pelvic antropometric measurement in 3D CT for placement of two unilateral iliosacral S1 - 7.3 mm screws

**DOI:** 10.1007/s00264-021-05095-1

**Published:** 2021-06-08

**Authors:** Arnold J. Suda, Lisa Helm, Udo Obertacke

**Affiliations:** 1 Department of Orthopaedics and Trauma Surgery, AUVA Trauma Center Salzburg, Academic Teaching Hospital of Paracelsus Medical University, Dr. Franz-Rehrl-Platz 5, 5010 Salzburg, Austria; 2 Department of Orthopaedics and Trauma Surgery, Medical Faculty Mannheim of Heidelberg University, University Medical Center Mannheim, Theodor-Kutzer-Ufer 1-3, 68167 Mannheim, Germany; 3 Department of Trauma and Orthopaedic Surgery, Asklepios Klinik Nord – Heideberg, Tangstedter Landstrasse 400, 22417 Hamburg, Germany

**Keywords:** Pelvis fractures, S1—screws, Unilateral screws, Measurement

## Abstract

**Purpose:**

Stability of the dorsal pelvic ring is important for patient mobilisation and can be restored using several surgical procedures after fracture. Placement of percutaneous iliosacral screws is a reliable and minimal-invasive technique to achieve stabilisation of the dorsal pelvic ring by placement of two screws in the first sacral vertebra. Aim of this study was to evaluate 3D CT scans regarding the anatomical possibility to place two 7.3 mm iliosacral screws for fixation of the dorsal pelvic ring.

**Methods:**

3D CT datasets of 500 consecutive trauma patients with 1000 hemipelves of a mid-european level I trauma centre with or without pelvic injury were evaluated and measured bilaterally in this retrospective study.

**Results:**

One thousand hemipelvic datasets of 500 patients (157 females, 343 males) with a mean age of 49.7 years (18 to 95) were included in this study. Only 16 hemipelves (1.6%, 11 in females, 5 in males) in 14 patients (2.8%, 9 females = 5.73%, 5 males = 1.5%) showed too narrow corridors so that 7.3 mm screw placement would not be possible (*p* = 0.001). In women, too narrow corridors occurred 3.9 times as often as in men. Only two females showed this bilaterally.

**Conclusion:**

The evaluation of 3D CT scans of the pelvis showed the importance of planning iliosacral screw placement, especially if two 7.3 mm screws are intended to be placed in the first sacral vertebra.

## Introduction

An intact dorsal pelvic ring is mandatory for human’s mobility with upright gait because of force transmission from spine, sacrum, iliosacral joints, and ilium to hip joints and lower extremities. Interruptions of the dorsal pelvic ring caused by fractures lead to severe pain and disability [1]. Previous studies have described safe corridors for iliosacral screw placemen [2–8]. Malpositioning of percutaneous screws remains a problem and can cause iatrogenic nerve injury or revision surgery [9, 10]. Flouroscopy in the emergency department includes one plain pelvic x-ray [11]. Diagnosis with this imaging is possible but not suitable for correct diagnosis and classifications or pre-operative planning. Further x-rays as inlet- or outlet-view are possible but 3D CT scan is gold-standard for these injuries [11, 12].

Several general pelvic-ring injury classifications have been introduced; the AO/OTA-classification and Young-Burgess classification are commonly used emphasizing on the dorsal ring injury as dorsal stability is clinically important [13, 14].

In definitive care, anatomical reduction and reconstruction should be achieved using external and/or internal stabilization. Unstable dorsal pelvic ring injuries should not be fixed with anterior external fixation only because of insufficient stability [15, 16].

Percutaneous screw fixation has low complication rates and small soft tissue trauma.

With CT-based navigation, a significant reduction of mal-positioning could be achieved [12, 17–20].

Two unilateral iliosacral screws perpendicular to the fracture line in the first sacral vertebra S1 reach the highest stiffness rates whereas one screw provides clinical sufficient stability [21–28]. Use of unilateral 7.3 mm cannulated screws is one option for fixation.

Biomechanical investigations showed that two unilateral screws in S1 generate less cut-out and allow more load cycles in the finite-element model [15, 29] and have less risk of neurological injury compared to screws in S2 [25, 30–34].

The aim of this retrospective study was to evaluate sacral S1 corridors in 1000 3D CT datasets to ensure pre-operative planning of unilateral double S1 iliosacral 7.3 mm screws with distance of 5 mm each in all patients independent of gender.

Following statements were hypothesized:

- The pelvic S1—canal is narrower in women
- The narrowest part of sacral S1—circle surface in sagittal view in CT-scan is suitable for positioning of two 7.3 mm iliosacral screws with distance of 5 mm in all patients.

To our knowledge, no other study has been published to evaluate this high number of measurements regarding gender-specific differences of unilateral positioning of two 7.3 mm iliosacral S1 screws.

## Methods

**Fig. 1:**
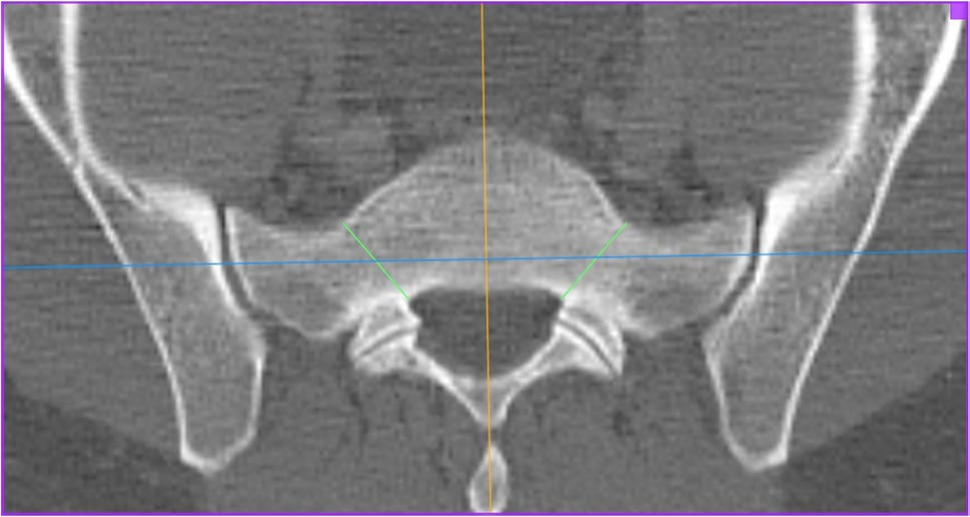
First sacral vertebral body in axial view

**Fig. 2:**
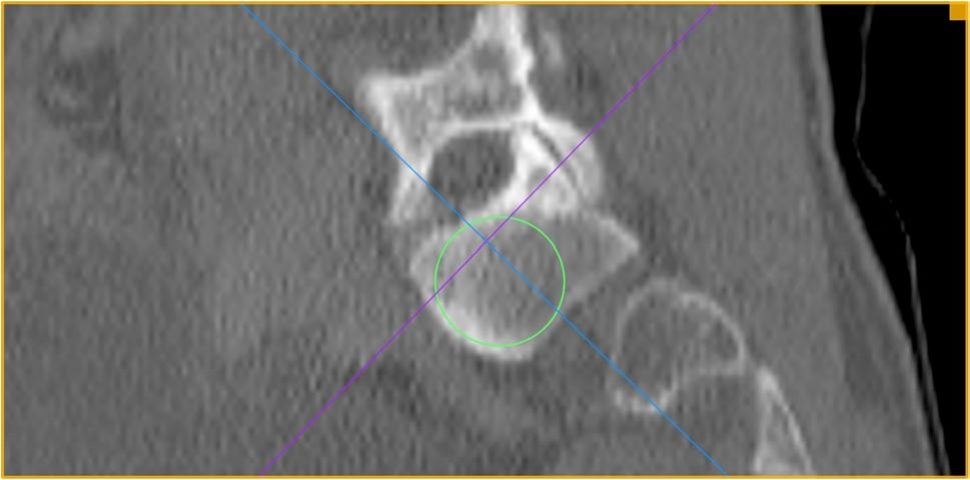
Cranial, caudal lower and upper plate with anterior cortex in sagittal view on both sides

**Fig. 3:**
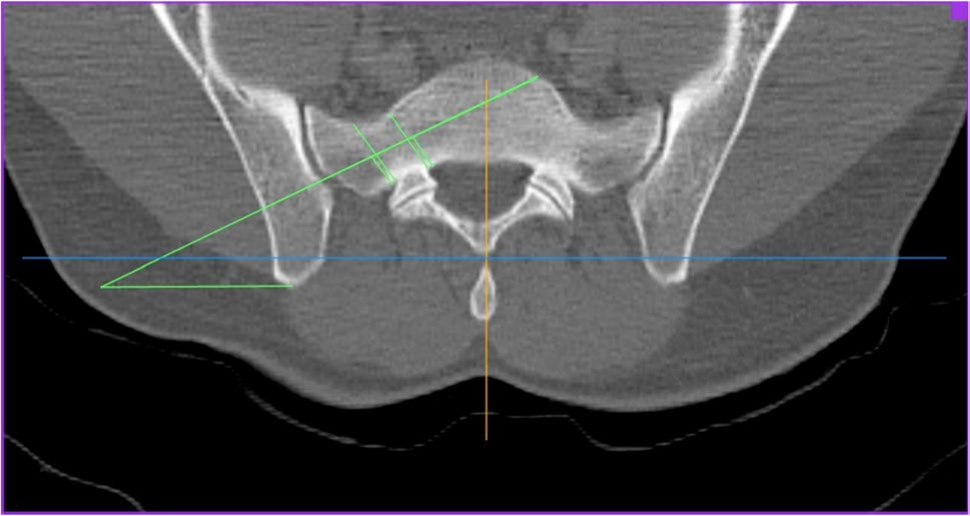
The level of the sacro-iliac joint in the axial view of both sides

The investigation was performed at the University Medical Center Mannheim, Medical Faculty Mannheim of Heidelberg University, Department of Orthopaedics and Trauma Surgery. After positive regional and institutional ethical committee vote (2016-870R-MA), bilateral evaluation and measurements of the 3D CT scan datasets of 500 consecutive trauma patients with datasets of 1000 hemipelves without pelvic trauma were performed by one single board-certified orthopaedic surgeon and validated by one board-certified radiologist. A mean measured value was calculated of the independent measurements of both investigators. Patients between 18 and 99 years of age were included in this study; patients with osteoporosis, healed pelvic or spinal injuries were not excluded. Patients with pelvic injuries or pathologies as tumors, the presence of implants, or anatomical disorders as lumbalization were excluded. The 3D CT scans were performed using a Siemens Somatom Sensation 16-slice multi-detection scanner using a 3 mm slice thickness. Measurements were carried out using the OsiriX DICOM Viewer (Pixmeo SARL, Switzerland) using one certified monitor. The following measurements were performed:

1. The narrowest part of the corridor from ala to corpus between foramen and anterior cortex, 0.4 mm below upper plate of the first sacral vertebral body in axial view (Fig. 1, green line).
2. The plane of the circle around the narrowest part of the corridor from ala junction to corpus between cranial and caudal lower and upper plate and anterior cortex in sagittal view on both sides (Fig. 2, green circle).
3. The length of the corridor crossing the narrowest part and the corridor angle parallel the tangent to both dorsal spinal processes at the level of the sacro-iliac joint in the axial view of both sides (Fig. 3, green line and green angle, respectively).

Eight variables were defined and calculated regarding sex (Table 1). Statistical analysis was performed using SPSS (SPSS Statistics for Windows, Version 24.0. Armonk, NY: IBM Corp.).

**Table 1:** Results of measurements

Variable	Values in cm				*p* value
	min	max	mean	SD	
Width of the narrowest part right and left axial, females	0.98	15.00	1.88	0.74	< 0.01
Width of the narrowest part right and left, axial, males	0.50	3.14	2.04	0.40	
Width of the narrowest part left, axial, females	0.98	15.00	1.90	1.12	= 0.09
Width of the narrowest part left, axial, males	0.58	3.14	2.05	0.41	
Height of the narrowest part, saggital, females	2.09	5.36	3.42	0.70	< 0.01
Height of the narrowest part, saggital, males	2.26	6.21	4.09	0.73	
Circle diameter narrowest part, sagittal, females	1.60	3.85	2.43	0.29	< 0.01
Circle diameter narrowest part, sagittal, males	1.89	5.29	2.63	0.36	

*min* minimum, *max* maximum, *SD* standard deviation

Hypothesis 1 was tested using Student’s *t* test with unpaired samples. The basis for this test was one quantitative dependent variable, two independent samples, normal distribution, unknown variance of the population, and sample homogeneity of variance. For evaluation of homogeneity of variance, the Levene test was performed. For hypothesis 2, evaluation was performed using single-sample *t* test because population’s variance was unknown and the sample size was > 30. With this test, significant deviation of mean values to test values was identified. Empiric mean value in this case is arithmetic mean of circle diameters. Test value (μ0 = 1.96 cm) is the summary of screw diameters and recommended distance between the screws. All subgroups showed variance homogeneity and *t* test could be performed with normally distributed data.

## Results

Five hundred patients (157 females, 343 males) with a mean age of 49.7 years (18 to 95) at time of investigation were included. The results are presented in Table 1 and show the circle surface of the narrowest part left and right sagittal significantly higher in males.

 Canals too narrow for placement of two 7.3 mm S1 screws were more common in women than in men: 5.7% of all females, 95% confidence interval CI: 4–12% and 5 males = 1.5% of all males; CI: 0.6–3.4%; *p* = 0.001.

Regarding the width of the narrowest left axial part, we did not find statistical significant differences between females and males.

In only 16 of 1000 hemipelves (1.6%, 11 in females, 5 in males) in 14 patients (2.8%, 9 females, 5 males), it would have been impossible to place two unilateral 7.3 mm screws with 5 mm distance to each other in the first sacral vertebra (ration females:males = 3.9:1). Only two patients showed this restriction bilaterally; in the other 12 cases, we found this unilateral (6 right, 6 left).

## Discussion

In our cohort, 5.7% of females but only 1.5% of males showed too narrow corridors for theoretical placement of double ipsilateral 7.3 mm S1 screws. Narrow corridors can be easily identified on preoperative CT scan with multiplanar reconstructions.

Except width of the narrowest sacral left axial part, all other parameters showed statistically significant number of narrower canals in females. In 98.4% of the patients, placement of two unilateral S1 7.3 mm screws would have been possible with 5 mm between the screws. In only 14 patients (16 measurements, 1.6%), the circle surface diameter was too narrow. We found a ratio of females:males of 3.9:1 for corridors which were too narrow.

Minimal-invasive percutaneous application of iliosacral screws using planar fluoroscopy is gold-standard in surgical treatment of iliosacral injuries. Awareness of anatomical landmarks and surgical technique is mandatory. Van Zwienen et al. showed in a biomechanical study that a second iliosacral screw in S1 raises rotational stability and load cycles in the finite-element-analysis plus delays failure which supports the rationale of our study [15]. In addition, two screws in S1 lower the risk of neurological injuries compared to positioning another screw in S2 [21–28, 30–32]. Female sex, advanced age, a high BMI, and previous child birth have previously been associated with narrow S1 and S2 canals [2, 35–41].

In our study, a high gauge of repeatability and reproducibility could be achieved through determination of landmarks in three planes of the dorsal pelvic ring in addition with the narrowest part of the S1—circle surface in sagittal view.

Our study shows that there are statistically significant gender-related differences in the safe corridors but in 97.2% of the patients (98.4% of all hemipelves), positioning of two unilateral iliosacral S1—7.3 mm screws would have been possible.

Bilaterally narrow corridors were uncommon in our collective: if bilateral S1 screw-fixation is planned, the risk of encountering a narrow corridor almost doubles.

This study has several limitations:

- First, this was a retrospective study in one single centre with one principal investigator and one validator.
- Second, only mid-European white patients have been included and the ratio of females:males was 1:2.2.
- Third, we did not evaluate information on low body height as risk factor for narrow canals.
- Fourth, with the measurement software used, clinical and intra-operative setting is not equally figured due to different scanners and viewers which could lead to failure or mismatch.
- Fifth, the CT scanner and the slice thickness of 3 mm are not used by every hospital which could affect the measurement in clinical practice. All measures are only a recommendation for pre-operative planning and support surgical procedure.

In 99% of male and in 96% of female hemi-pelvises in this central European cohort, there was enough room to place two 7.3 mm screws at 5 mm distance into the S1 vertebra.

Patients with too narrow corridors can be identified in 3D CT scan preoperatively and therapy can be adapted to anatomical conditions. In a future study, elderly patients with low-energy pelvis trauma will be included to re-evaluate the findings of this study.

## Data Availability

With the corresponding author due to ethical committee vote.
